# Different osteosyntheses for Colles' fracture: A mechanical study in 42 cadaver bones

**DOI:** 10.3109/17453670902947440

**Published:** 2009-04-01

**Authors:** Yngvar Krukhaug, Nils R Gjerdet, Odd J Lundberg, Peer K Lilleng, Leiv M Hove

**Affiliations:** ^1^Department of Orthopaedic Surgery, Haukeland University Hospital, Helse-Bergen HFBergenNorway; ^2^Department of Clinical Dentistry - Biomaterials, University of BergenBergenNorway; ^3^Department of Surgical Sciences, University of BergenBergenNorway; ^4^Department of Surgical Sciences, the Gade Institute, Section for Pathology, University of BergenBergenNorway

## Abstract

**Background and purpose** In recent years several different plate designs for internal fixation of fractures of the distal radius have been developed. However, few biomechanical studies have been performed to compare these new implants. The purpose of this study was to compare the mechanical properties of 5 different commercially available plates (3 volar and 2 dorsal) with standard K-wire fixation using a distal radial cadaver model.

**Material and methods** 42 human radial bones from 26 cadavers were included. The bone mineral density (BMD) was measured by DEXA in all bones, and the radial bones were assigned to 6 equiv alent groups based on bone density and total amount of mineral. A distal radial osteotomy was done and a dorsal 30-degree wedge of bone was removed. 1 K-wire fixation group and 5 plate groups were tested for rigidity, yield load, and maximum load.

**Results** When data from dorsally and volarly applied plates were pooled, we did not find any statistically significant differences between them regarding stiffness, yield load, and maximum load. The K-wire group showed significantly lower yield load than 3 of the plate groups. There were no statistically significant differences in yield load between the 5 plate groups. The K-wire group showed lower rigidity than the plate groups. The K-wire group and 1 plate group failed at a statistically significant lower maximum load than the 4 other plate groups.

**Interpretation** The volar plates had the same mechanical stability as the dorsally applied plates, and they are therefore a good alternative to dorsally applied plates. K-wire osteosynthesis was inferior to plate osteosyntheses regarding all mechanical properties.

## Introduction

For many years dorsal plate osteosynthesis was the gold standard for fractures of the distal radius, if plate osteosynthesis was considered. However, complications such as tendinitis and ruptures of extensor tendons secondary to direct contact with the plates were not uncommon (Ring et al. 1997, Carter et al. 1998, Kambouroglou and Axelrod 1998, Lowry et al. 2000). Thus, many authors have recently advocated volar plating—even for dorsally displaced fractures (Kamano et al. 2002, Orbay and Touhami 2006).

We compared the mechanical behavior of fractures of the distal radius that were fixed with different plates in cadaver bones. Pin osteosynthesis was used as a reference. All the plates were made of stainless steel and were made by the same manufacturer.

## Material and methods

### Specimen preparation

**Figure 1.**
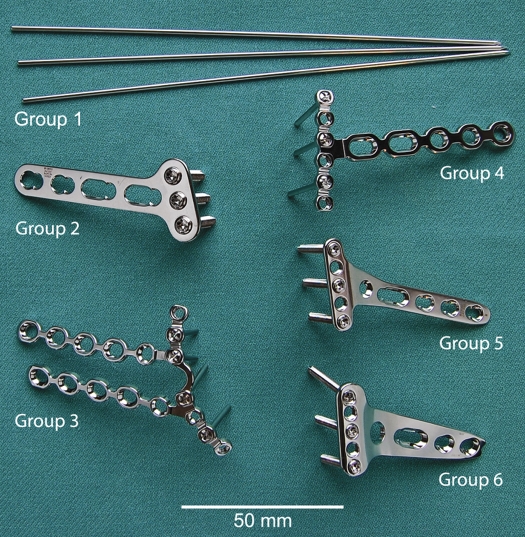
The implants. Group 1: three 1.6-mm K-wires. Group 2: Synthes stainless steel 3.5-mm dorsal locking T-plate. Group 3: AO Synthes stainless steel Dorsal Distal Radius Plate. Group 4: Synthes stainless steel Volar Distal Radius Plate. Group 5: Synthes LCP Distal Radius Plate 2.4-mm, volar. Group 6: Synthes LCP Buttress Plate 2.4-mm for distal radius, volar.

We selected the distal 12 cm of the radius from 42 fresh frozen cadaver bones with no bone deformities or signs of prior fractures; this was stripped of all soft tissue. The 42 specimens had been taken from 26 corpses, median age 53 (range 19–73, quartile 46–61) years. There were 27 specimens from male cadavers and 15 from females. For all 42 specimens, bone density and total amount of mineral in the distal radius were measured with dual-energy X-ray absorptiometry (DEXA). The radial bones were arranged into six similar groups, based on total amount of mineral and density (Table 1). The groups were then randomized to 1 of 6 different osteosyntheses according to the following list (see also Figure 1):

**Table 1.** Median total amount of mineral in and mean bone density of the distal radius

	Mean total amount of mineral, g (range) (SD)	Mean bone density, g/cm^2^ (range) (SD)
Group 1	1.6 (0.9–2.6) (0.61)	0.34 (0.21–0.51) (0.11)
Group 2	1.8 (0.9–2.2) (0.55)	0.32 (0.17–0.44) (0.09)
Group 3	1.8 (0.4–2.7) (0.74)	0.32 (0.08–0.54) (0.14)
Group 4	1.7 (1.0–2.6) (0.57)	0.33 (0.22–0.51) (0.09)
Group 5	1.7 (1.0–2.2) (0.51)	0.32 (0.23–0.41) (0.07)
Group 6	1.7 (1.0–2.1) (0.43)	0.31 (0.22–0.41) (0.08)

**Figure 2.**
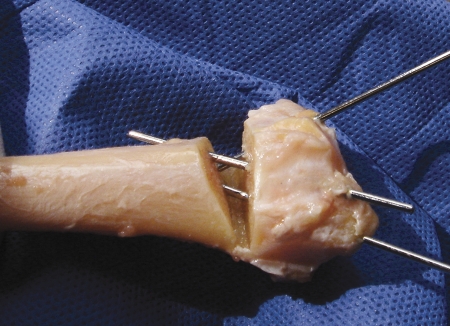
The placement of the 3 K-wires.

Group 1: Three 1.6-mm K-wires. 2 wires were placed in the radial styloid and secured in the ulnar cortex, 1 volar and 1 dorsal. 1 wire was placed in the ulnar aspect of the distal fragment and secured in the radial cortex (Figure 2). Group 2: Synthes stainless steel 3.5-mm dorsal locking T-plate. Group 3: AO Synthes stainless steel Dorsal Distal Radius Plate (The pi-plate). Group 4: Synthes stainless steel Volar Distal Radius Plate. Group 5: Synthes LCP Distal Radius Plate 2.4 mm, volar. Group 6: Synthes LCP Buttress Plate 2.4 mm for distal radius, volar.

**Figure 3.**
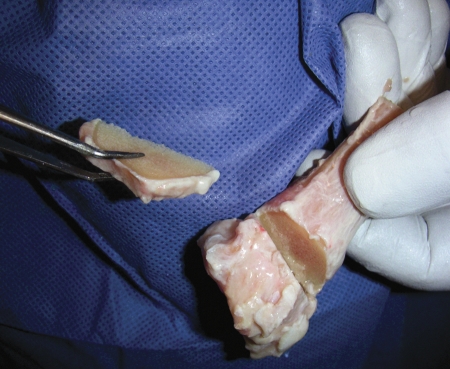
The osteotomy gap.

An osteotomy was made 15 mm proximal to the ulnar joint facet using a water-cooled saw. To mimic dorsal comminution, a dorsal 30-degree wedge of bone was taken out. The volar cortex was not osteotomized, but fractured without fragmentation (Figure 3). The plates were applied as recommended by the manufacturer.

**Figure 4.**
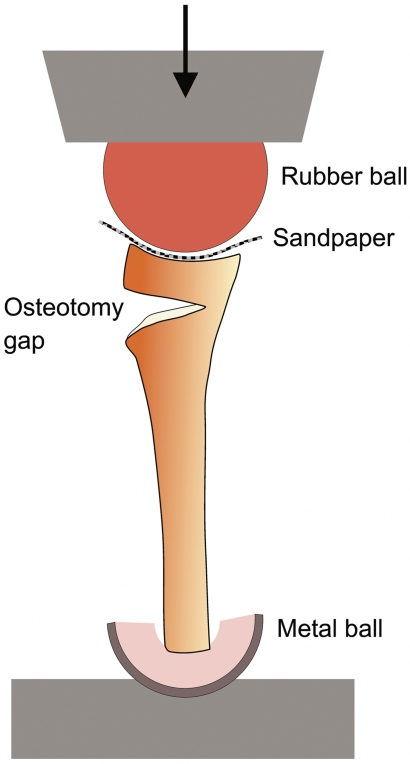
The test set-up.

**Figure 5.**
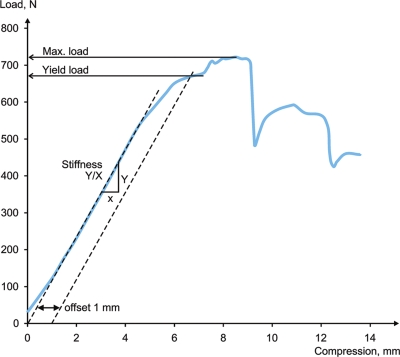
Typical load/deformation curve.

To avoid mechanical changes in the bones, the cadaver bones were thawed for 3 h at most before the osteotomies were performed and the mechanical tests were done. The specimens were tested using a specially designed compression set-up mounted in a servo-hydraulic mechanical testing machine (MTS 510; MTS Systems Corp., Eden Prairie, MN). The specimens were mounted vertically, allowing free rotation of the proximal end (Figure 4). The testing was performed with a deformation rate of 1 mm/s in the axial direction. Force and deformations were recorded continuously by a load cell and a differential transformer during the tests. Testing continued until failure or complete closure of the osteotomy gap. The resulting load/deformation data were evaluated with software that calculated the *rigidity* of the construction, taken as the slope of the line through the initial, almost straight portion of the load/deformation curve, the *yield load*, representing the load causing a 1-mm offset from the line described for determination of rigidity, and the *maximum load* (Figure 5).

The Bergen regional ethics committee approved the study (No.168.05).

### Statistics

Mixed linear models were used to account for 2 wrists from the same cadaver. The type of implant was included in the mixed model as a fixed (categorical) factor, while cadaver number was included as a random factor. SPSS software version 15.0 was used for all the statistical analyses. Values of p ≤ 0.05 were considered statistically significant.

## Results

All dorsal plate osteosyntheses failed with angulation, and in all cases the apex was pointing dorsally. The volar plate osteosyntheses and the K-wire osteosyntheses all failed, with the apex pointing volarly.

### Mechanical data

**Figure 6.**
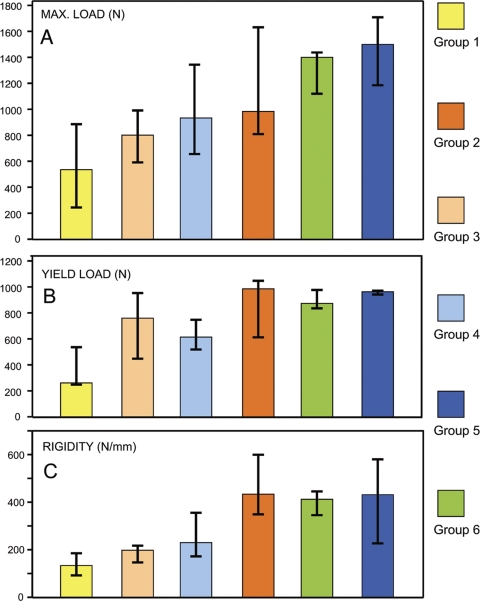
Means of the biomechanical variables measured. The whiskers represent standard deviation. A. Maximum load. B. Yield load. C. Rigidity. In all groups, n = 7 except for the maximum load for the pin group and the LCP Distal Radius plate group, where n = 6.

The average yield load (N), rigidity (N/mm), and maximum load (N)—based on 7 specimens in each group—are shown in Figure 6. It was not possible to record the maximum load for 1 specimen in group 1 and for 1 in group 6 because the specimens exhibited a continuous compression without passing through a maximum.

### Yield load

The K-wire group (group 1) showed lower yield load than any of the plate groups. The mean differences reached statistical significance in 3 of the plate groups (group 1 vs. group 2: p = 0.004; group 1 vs. group 5: p = 0.005; and group 1 vs. group 6: p = 0.004). There were no statistically significant differences in yield load between each of the 5 plate groups (Table 2).

**Table 2.** Mean differences in the mean yield load, with 95% CI in parentheses

	Group 1	Group 2	Group 3	Group 4	Group 5	Group 6
Group 1	–	584 (203, 964) **^a^**	362 (–15, 739)	319 (–53, 691)	550 (179, 921) **^a^**	567 (191, 944) **^a^**
Group 2			–221 (–596, 152)	–264 (–642, 112)	–34 (–414, 347)	16 (–400, 368)
Group 3				–43 (–420, 334)	188 (–187, 562)	205 (–172, 582)
Group 4					231 (–143, 605)	248 (–124, 621)
Group 5						17 (–363, 397)
Group 6						–

**^a^** p ≤ 0.05.

There was a correlation of 0.39 between total amount of mineral and yield load (p = 0.01). The slope in linear regression was 279 per unit change in total amount of mineral.

There was no statistically significant difference in yield load between the volar and dorsal plate groups when the data were pooled.

### Rigidity

The K-wire group showed lower rigidity than the plate groups. The mean differences were statistically significant for 3 of the plate groups (group 1 vs. group 2: p = 0.001; group 1 vs. group 5: p = 0.02; and group 1 vs. group 6: p = 0.03) (Table 3).

**Table:** **Table 3.**Mean differences in mean stiffness, with 95% CI in parentheses

	Group 1	Group 2	Group 3	Group 4	Group 5	Group 6
Group 1	–	364 (150, 578) **^a^**	67 (–147, 281)	170 (–44, 383)	270 (56, 484) **^a^**	247 (33, 461) **^a^**
Group 2			–297 (–511, –83) **^a^**	–194 (–408, 20)	–94 (–308, 120)	–117 (–331, 97)
Group 3				103 (–111, 317)	203 (–11, 417)	180 (–34, 394)
Group 4					100 (–114, 314)	77 (–137, 291)
Group 5						–23 (–237, 191)
Group 6						–

**^a^** p ≤ 0.05.

The pi-plate (group 3) was also found to have lower mean rigidity (p= 0.008) than the 3.5-mm dorsal locking T-plate (group 2). There was no statistically significant correlation between the total amount of bone mineral and rigidity (p = 0.2). There was no statistically significant difference in rigidity between the volar and dorsal plate groups when the data were pooled.

### Maximum load

The K-wire group (group 1) failed at a lower mean maximum load than 4 of the 5 plate groups (group 1 vs. group 2: p = 0.01; group 1 vs. group 4: p = 0.05; group 1 vs. group 5: p < 0.001; and group 1 vs. group 6: p = 0.003) (Table 4).

**Table:** **Table 4.** Mean differences in mean maximal load, with 95% CI in parenthese**s**

	Group 1	Group 2	Group 3	Group 4	Group 5	Group 6
Group 1	–	627 (152, 1,104) **^a^**	239 (–229, 707)	453 (2, 904) **^a^**	957 (496, 1417) **^a^**	740 (276, 1,204) **^a^**
Group 2			–389 (–832, 54)	–175 (–624, 275)	329 (–147, 805)	112 (–352, 577)
Group 3				214 (–235, 663)	718 (257, 1,179) **^a^**	501 (52, 951) **^a^**
Group 4					503 (45, 962)	287 (–147, 721)
Group 5							–216 (–689, 257)
Group 6							–

**^a^** p ≤ 0.05.

The pi-plate (group 3) failed at lower mean maximum load than 2 of the other plate groups (group 3 vs. group 5: p = 0.003; group 3 vs. group 6: p = 0.03).

There was a positive correlation of 0.43 between total amount of mineral and maximum load (p = 0.006). The slope in linear regression was 396 per unit change in total amount of mineral.

When comparing other groups according to yield load, rigidity, and maximum load the differences did not reach statistically significant levels. There was no statistically significant difference in maximum load between the volar and dorsal plate groups when the data were pooled.

## Discussion

The cadaver bones had been stripped of soft tissue, frozen at –80ºC, and thawed before DEXA measurements. This means that measurements cannot be directly compared to values found in living individuals. However, the cadaver bones without soft tissue did allow us to compare the different specimens used in the study. The strength and elastic modulus of the bone do not deteriorate significantly in the process of freezing (Linde and Sorensen 1993).

We found that there was a correlation between high bone density and maximum load. The risk of fracture increases with reduced bone density (BMD), and there is a higher incidence of loss of reduction in distal radius fractures in patients with osteoporosis (Dias et al. 1987, Earnshaw et al. 1998).

We found that K-wire osteosynthesis had worse mechanical properties than plate osteosynthesis. Another mechanical study on cadaver bones with dorsaly placed osteotomies has shown that dorsal plates are mechanically more stable than volar plates (Blythe et al. 2006). In our study, however, we did not find any statistically significant mechanical differences between the volar and dorsal plates. The fixed-angle volar plates (groups 5 and 6) could take higher loads than the dorsal T-plates (group 2). On the other hand, dorsal double plating with two 2.0-mm plates has been assumed to be mechanically superior to one pi-plate or one T-plate (Peine et al. 2000). We tested systems with only 1 plate applied.

Yield load appears to be more clinically relevant than maximum load regarding stability of the osteosynthesis. The selection of a yield limit (offset) of 1 mm indicates a permanent deformation of clinical concern as the osteosynthesis starts to fail. The maximum load (failure load) may reflect the quality of the bone rather than the performance of the osteosynthesis.

A possible limitation of our study may have been the extraarticular model we used. The results cannot be applied directly to intraarticular fractures. However, we used the extraarticular model because it is difficult to obtain a standardized intraarticular fracture model of the distal radius. By creating a fracture model with the volar cortex fractured but not fragmented (AO type A3), we simulated the most stable of all unstable situations (Drobetz et al. 2006). Intraarticular fractures and fractures with volar comminution are much more unstable. This probably means that the differences between the methods of fixation will increase when used in more complex fractures.

The bending strength (rigidity) of a construction with a plate placed on the tension side of a bone is about 100 times higher when there is bone contact on the compression side (Cochran 1982). Because of this, it is likely that the strength of the dorsal plates (the Synthes stainless steel 3.5-mm dorsal locking T-plate (group 2), the AO Synthes stainless steel Dorsal Distal Radius Plate (the pi-plate, group 3) and the K-wire (group 1) decreased substantially in fractures where the volar cortex is comminuted. Similarly, the strength of the volar plates might increase in Smith'stype fractures where the dorsal cortex is not fragmented. Another limitation of our study is that the wrists were stripped of all soft tissue, and the force used to test the constructs was applied in the axial direction of the radial shaft. This is an unphysiological situation; nor were the constructions tested with cyclical loads, which would have been a more physiological set-up.

In recent years, several implants have been introduced that are applied volarly even when the fractures have dorsal comminution. This means that the new volar implants probably need to be stronger to avoid loss of reduction. We found, however, that 2 of the 3 volar plates (Synthes LCP Distal Radius Plate 2.4-mm volar (group 5) and Synthes LCP Buttress Plate 2.4-mm for distal radius, volar (group 6)) withstood more load before failure than the dorsal plates.

The modes of failure reflect the overall characteristics of the bone/plate construct. All the dorsal plate osteosyntheses failed in apex dorsal angulation, and volar plate osteosyntheses and the K-wire failed in apex volar angulation. In all cases, the plate bending occurred without loosening of screws. The common situation (seen clinically) is that fractures treated with plate fixation fail due to screw loosening and/or displacement. The reason that this phenomenon did not occur in our experiment may have been the axial load. In clinical situations, there are cyclic loads in different directions. Our results should therefore be interpreted with caution.

Due to our relatively small series, one must also keep in mind that statistically insignificant findings of differences between the plate groups represent absence of evidence for differences, and not evidence for absence of differences.

In summary, our findings indicate that volar locking plate osteosynthesis is a good alternative to the dorsally applied plates in distal radius fractures.
